# Experimental Adaptation of Wild-Type Canine Distemper Virus (CDV) to the Human Entry Receptor CD150

**DOI:** 10.1371/journal.pone.0057488

**Published:** 2013-03-12

**Authors:** Maria Bieringer, Jung Woo Han, Sabine Kendl, Mojtaba Khosravi, Philippe Plattet, Jürgen Schneider-Schaulies

**Affiliations:** 1 Institute for Virology and Immunobiology, University of Würzburg, Würzburg, Germany; 2 Department for Clinical Veterinary Medicine, Vetsuisse Faculty, University of Bern, Bern, Switzerland; Thomas Jefferson University, United States of America

## Abstract

Canine distemper virus (CDV), a close relative of measles virus (MV), is widespread and well known for its broad host range. When the goal of measles eradication may be achieved, and when measles vaccination will be stopped, CDV might eventually cross the species barrier to humans and emerge as a new human pathogen. In order to get an impression how fast such alterations may occur, we characterized required adaptive mutations to the human entry receptors CD150 (SLAM) and nectin-4 as first step to infect human target cells. Recombinant wild-type CDV-A75/17^red^ adapted quickly to growth in human H358 epithelial cells expressing human nectin-4. Sequencing of the viral attachment proteins (hemagglutinin, H, and fusion protein, F) genes revealed that no adaptive alteration was required to utilize human nectin-4. In contrast, the virus replicated only to low titres (10^2^ pfu/ml) in Vero cells expressing human CD150 (Vero-hSLAM). After three passages using these cells virus was adapted to human CD150 and replicated to high titres (10^5^ pfu/ml). Sequence analyses revealed that only one amino acid exchange in the H-protein at position 540 Asp→Gly (D540G) was required for functional adaptation to human CD150. Structural modelling suggests that the adaptive mutation D540G in H reflects the sequence alteration from canine to human CD150 at position 70 and 71 from Pro to Leu (P70L) and Gly to Glu (G71E), and compensates for the gain of a negative charge in the human CD150 molecule. Using this model system our data indicate that only a minimal alteration, in this case one adaptive mutation, is required for adaptation of CDV to the human entry receptors, and help to understand the molecular basis why this adaptive mutation occurs.

## Introduction

The genus Morbilliviruses of the *Paramyxoviridae* family comprises the strictly human pathogen measles virus (MV), as well as animal infectious agents such as rinderpest virus (RPV), canine and phocine distemper viruses (CDV and PDV), peste des petits ruminants virus (PPRV), and the cetacean dolphin and porpoise Morbilliviruses (DMV and PMV). These viruses are all highly contagious and cause systemic infections resulting in devastating diseases in their respective hosts. Remarkably, all Morbilliviruses except CDV have a tightly restricted host range, whereas CDV has a broad host range infecting many carnivores including dogs, wolves, hyena, foxes, raccoons and ferrets. In addition, CDV strains substantially differ in virulence [1]. Expansions of the CDV host range took place several times during the last decades. For example, CDV from wolves, foxes, or dogs caused an epidemic among seals in the Lake Baikal and the Caspian Sea, and also a devastating disease within the lion population in the Serengeti [2], [3], [4]. Moreover, infections of monkeys (*Macaca fuscata* and *Macaca mulata*) with CDV resulting in quite high case fatality rates have been observed [5], [6], [7], [8]. During the recent outbreaks in monkey breeding farms in China approximately 10,000 animals were infected (25–60% disease incidence) and 5–30% of these died [5].

These findings suggest that CDV may eventually infect humans and spread in the human population. Indeed, specific anti-CDV antibodies indicating a former CDV-infection have rarely been detected in humans [9]. However, since antibodies generated in dogs against the MV-vaccine provide effective cross-protection against CDV [10], and since human sera containing anti-MV antibodies cross-react with CDV, it is widely accepted that measles-immunity protects humans at least partially from CDV infections. In spite of these findings it should not be excluded that CDV might eventually infect humans by escaping from the MV-vaccine-induced immunity, or when vaccination might be stopped after eradication of measles, which is now aspired by the World Health Organization (WHO) to be achieved until 2020 [11]. Because of this potential threat for the human population it is important to know how easy the species barrier to humans may be crossed.

We here investigated the requirements of wild-type CDV adaptation to the cognate human cellular MV receptors CD150 and nectin-4 [12], [13], [14], [15], [16], [17]. We found that adaptation of CDV to nectin-4 required none, and adaptation to CD150 only one amino acid exchange in the viral H protein. This adaptation process swiftly occurred within only three virus passages. The detected sequence alteration in the CDV-H protein reflects the molecular changes between canine and human CD150.

## Materials and Methods

### Cell lines and viruses

The human caucasian brochioalveolar carcinoma cell line NCI-H358 was obtained from Sigma-Aldrich (No. 95111733) and grown in RPMI 1640 medium containing 10% fetal calf serum (FCS). Vero cells expressing human CD150 (Vero-hSLAM) and dog (canine) CD150 (Vero-cSLAM) were a gift of Dr. Y. Yanagi, Fukuoka, Japan [18], [19]. They were cultured in Eagle's minimal essential medium (EMEM) containing 5% FCS, 100 U/ml penicillin and 100 µg/ml streptomycin. Recombinant wild-type CDV-A75/17^red^ [20], [21], [22] was propagated using Vero cells expressing canine CD150 (Vero-cSLAM).

### Virus preparation and titrations

Virus was harvested from 25 cm^2^ cell culture flasks by standard freezing and thawing of infected cell monolayers (with 1 ml medium/25 cm^2^ flask), scraping off the cells, homogenization, and removal of cell debris by 15 min centrifugation at 4000 rpm. Plaque assays were performed in triplicates in 6 well plates as described earlier. Briefly, 70% confluent receptor-expressing Vero cell monolayers were infected with a tenfold dilution series of the virus in EMEM containing 0% FCS. Further incubation at 37°C for 1–2 hour was followed by overlaying the wells with 3 ml of double EMEM supplemented with 5% FCS and 2% Noble agar (Sigma). Plaques were visualized after 6 days of incubation at 37° by staining with neutral red.

### Antibody infection inhibition assay

Vero-hSLAM cells were incubated with anti-human CD150 monoclonal antibody (mAb A12; BD Pharmingen) in 0% FCS Medium for 3 h at room temperature. As isotype control purified mouse IgG1k (clone MOPC-31C; BD Pharmingen) was used. Antibody treated target cells were then infected with virus at a MOI of 0.1 for 3 h before the medium was changed to FCS containing medium and incubated further at 37°C. Fluorescence and bright field pictures were taken at the respective time points.

### RNA isolation, RT-PCR and sequencing

Total RNA from infected cells was prepared using Trizol (Invitrogen) according to the manufacturer's protocol. RT was performed with random priming followed by PCR amplification of CDV fragments using specific primers. PCR-fragments were purified using QIA quick Gel Extraction Kit (QIAGEN) and sequenced by dideoxynucleotide chain termination (ABI Prism) using Big Dye® Cycle Sequencing Kit version 3.1. The primers used for amplification and sequencing are available upon request.

### CDV-H and –F expression plasmids and site directed mutagenesis

Vero-cSLAM cells, in 6-well plates at 90% confluency, were co-transfected with 2 µg of standard pCI-CDV-F_A75/17_ construct, and 1 µg of pCI-CDV-H_A75/17_ plasmid or derivative mutant with 9 µl of Fugene HD (Roche), according to the manufacturer's protocol. Phase contrast pictures were taken 24 h post-transfection by microscope (Olympus, Fluoroview, FV1000). The single substitution D540G was introduced by changing the corresponding GAC to GGC in pCI-CDV-H_A757/17_ using the quick-change lightning site-directed mutagenesis kit (Stratagene) resulting in plasmid pCI-CDV-H_A75/17-D540G_.

### Structural modelling

Structural modelling was performed using the program Phyre2 and protein structure prediction on the web [23] and 3DLigandSite (Imperial College, London) [24].

## Results

### CDV attachment proteins can utilize human nectin-4 without adaptive alterations

For experimental adaptations described in this manuscript we used CDV-A75/17^red^, a recombinant virulent wild-type CDV, which was originally isolated from the brain of a CDV-infected dog, cloned as a recombinant virus expressing tD tomato red fluorescent protein from the 4^th^ expression cassette, and propagated using Vero-cSLAM cells [20], [21], [22]. In order to adapt CDV-A75/17^red^ to human nectin-4 we used the human epithelial cell line H358 which is a target cell for measles virus expressing nectin-4, but not CD150 [16], [17]. The expression of nectin-4 and absence of CD150 was confirmed by FACS analysis (not shown).

**Figure 1:**
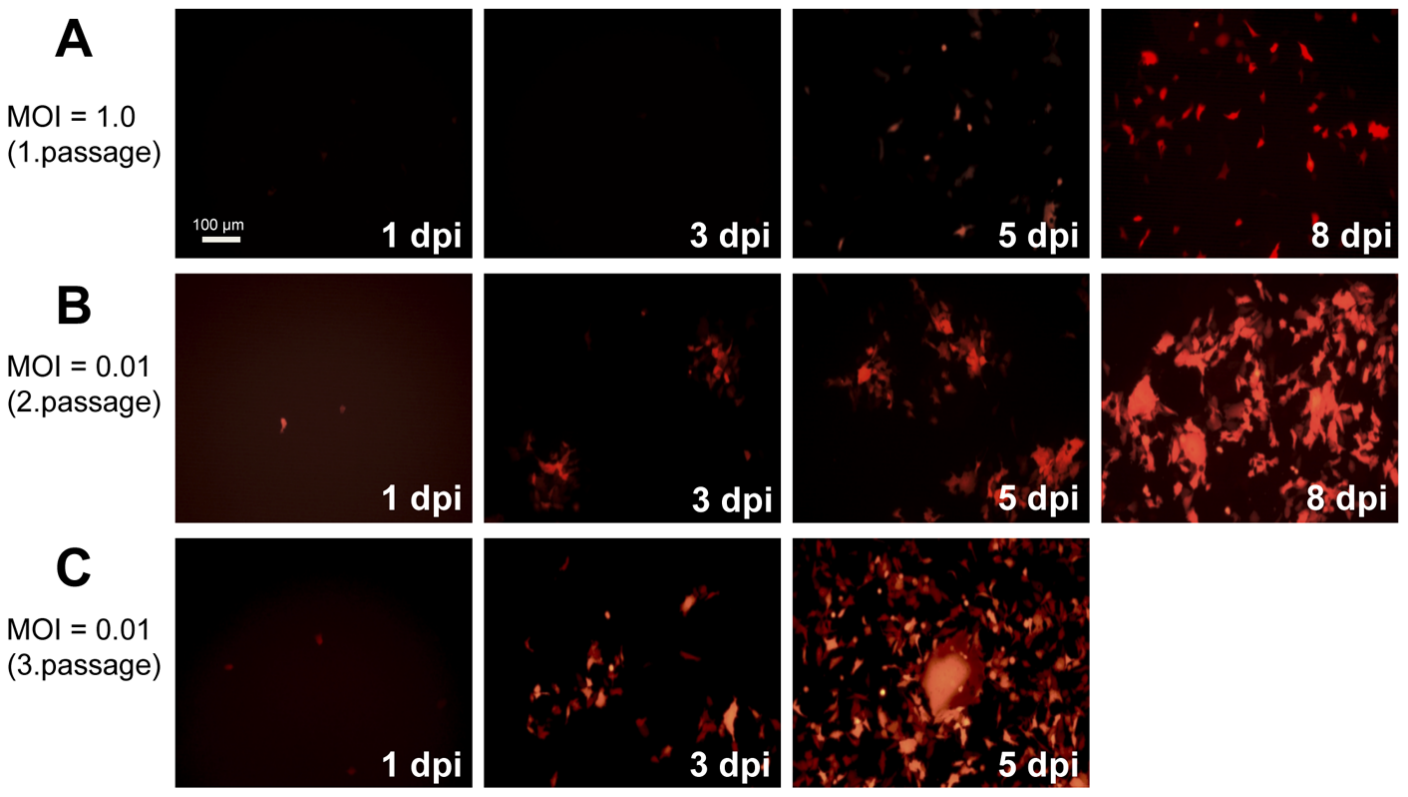
Infection of human epithelial cells H358 with CDV-A75/17^red^. H358 cells were infected with recombinant wild-type CDV-A75/17^red^ at a MOI of 1.0 (**A**), incubated for the indicated times and pictures of the red autofluorescence taken under UV light. At 8 days post-infection (dpi) virus was prepared and titrated using Vero-cSLAM cells, and H358 cells infected with this virus at a MOI of 0.01 (**B**). After 8 dpi virus was again prepared and titrated using Vero-cSLAM cells, and H358 cells were infected with this virus at a MOI of 0.01 for indicated times (**C**). Photomicrographs of the autofluorescent virus-encoded tD tomato red were taken under UV light (bar = 100 µm).

Human H358 cells were initially infected with CDV-A75/17^red^ at a MOI of 1.0 and incubated for the indicated times (Fig. 1 A). At 8 days post-infection (dpi), when a considerable number of individual infected cells were visible, virus was prepared and titrated using Vero-cSLAM cells (1^st^ passage of virus). H358 cells were then infected with this virus at a MOI of 0.01 (Fig. 1 B). At 8 dpi virus was again prepared and titrated using Vero-cSLAM cells (2^nd^ passage). H358 cells were then infected with this virus at a MOI of 0.01 (Fig. 1 C). Due to the advanced infection virus was harvested already at 5 dpi (3^rd^ passage) and titrated using Vero-cSLAM cells. The viral titres that were determined for each passage increased from 10^4^ (1^st^ passage) to 10^5.5^ pfu/ml (3^rd^ passage), but did not further increase in following passages. This 3^rd^ passage virus was then completely sequenced. We found mutations outside of the H and F genes, but no sequence alteration in the H and F genes in comparison to the parental recombinant CDV-A75/17^red^ indicating that no alteration of H is required for adaptation to human nectin-4.

### Adaptation of wild-type CDV-A75/17^red^ to human CD150 (SLAM)

**Figure 2:**
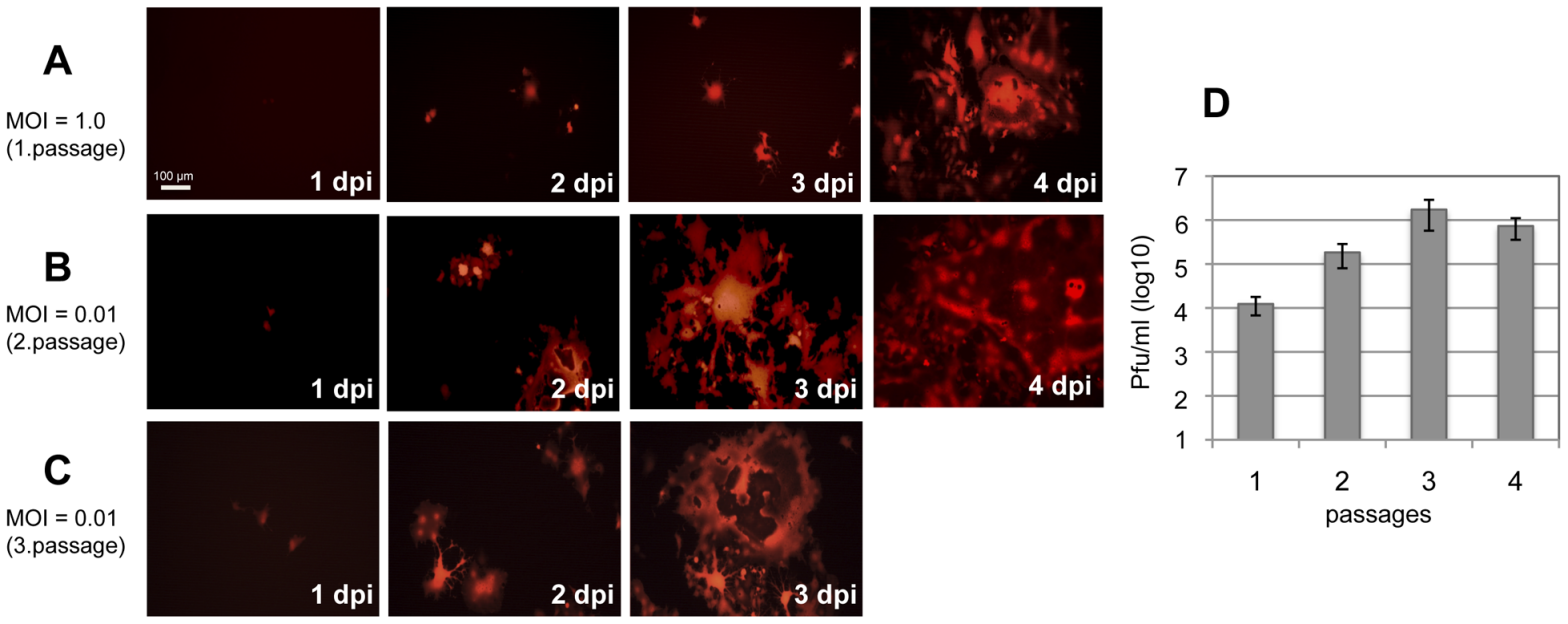
Infection of Vero-hSLAM cells with CDV-A75/17^red^. Human CD150-expressing Vero cells (Vero-hSLAM) were infected with recombinant wild-type CDV-A75/17^red^ at a MOI of 1.0 (**A**), incubated for the indicated times and pictures of the red autofluorescence taken under UV light. At 4 days post-infection (dpi) virus was prepared and titrated, and Vero-hSLAM cells infected at a MOI of 0.01 (**B**). After 4 dpi virus was again prepared and titrated, and Vero-hSLAM cells were infected with this virus at a MOI of 0.01 for indicated times (**C**). Titres of the virus preparations were determined using Vero-cSLAM cells and are given in (**D**). Photomicrographs of the autofluorescent virus-encoded tD tomato red were taken under UV light (bar = 100 µm).

Human CD150-expressing Vero cells (Vero-hSLAM) were initially infected with CDV-A75/17^red^ at a MOI of 1.0 (Fig. 2 A). After 4 days, when large syncytia were visible, virus was harvested (1^st^ passage). Virus was titrated using Vero-cSLAM cells, and Vero-hSLAM cells were infected at a MOI of 0.01 (Fig. 2 B). After 4 dpi when large syncytia were observed virus was prepared and again titrated using Vero-cSLAM cells (2^nd^ passage). Vero-hSLAM cells were then infected with this virus at a MOI of 0.01 (Fig. 2 C) and harvested at 3 dpi (3^rd^ passage). The corresponding titres of these virus passages as determined at day 4 (1^st^ and 2^nd^ passage) or day 3 (3^rd^ and 4^th^ passage) using Vero-cSLAM cells are given in (Fig. 2 D). Further passages of virus were produced accordingly. In the first 3 passages viral titres increased to approximately 10^5.5^ pfu/ml. In later passages virus titres did not further increase. This adaptation process was independently repeated three times using original recombinant wild-type virus. In all trials the swift adaptation within three passages was reproduced. The adapted virus formed similar syncytia with Vero-hSLAM cells as the parental virus with Vero-cSLAM cells demonstrating that receptor interactions including fusion helper functions appear to be fully adapted.

**Figure 3:**
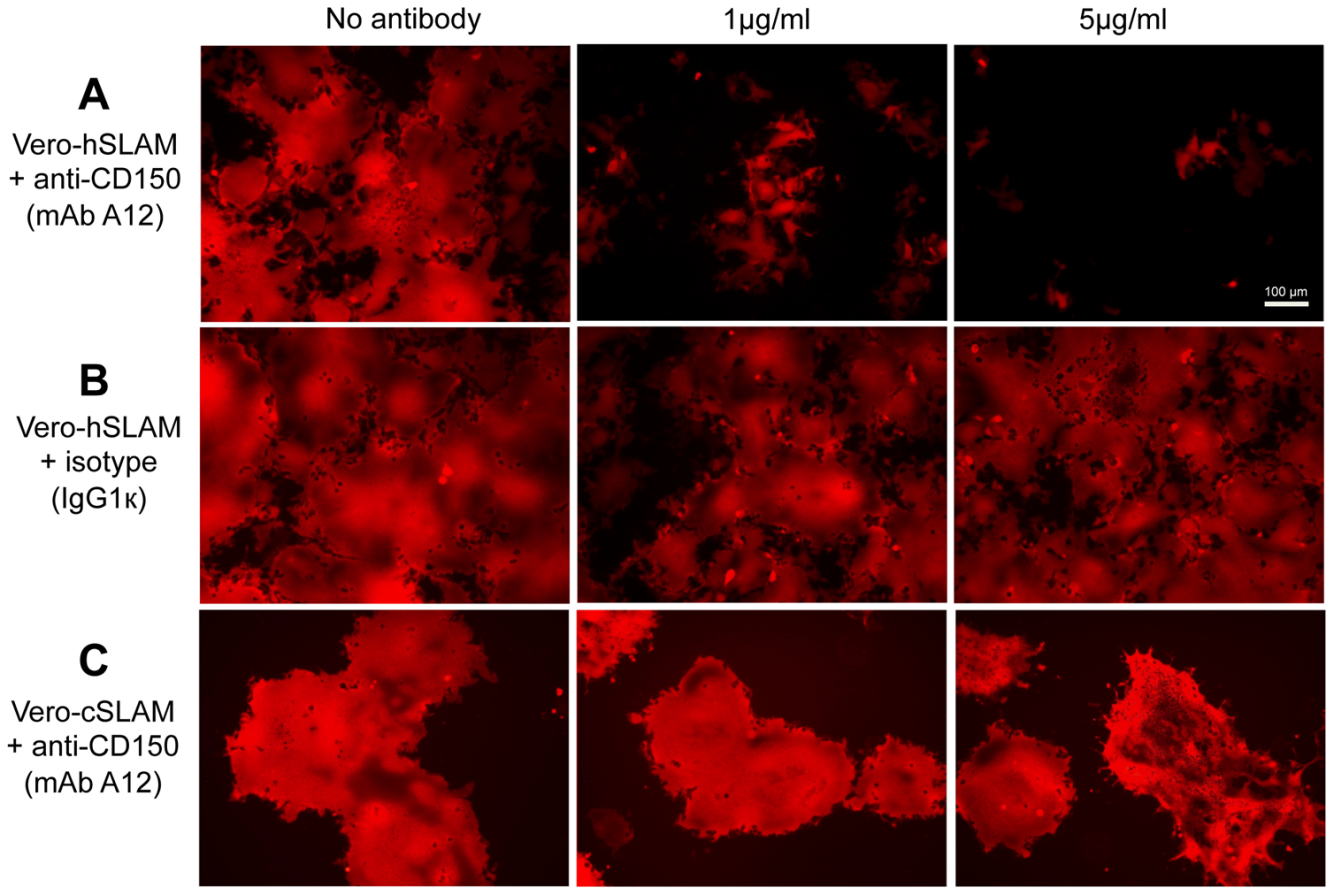
Infection with human CD150-adapted CDV-A75/17^red^ is specifically inhibited by CD150 antibodies. Vero-hSLAM cells were infected in the absence and presence of increasing concentrations of human CD150-specific monoclonal antibody A12 (**A**; 1 and 5 µg/ml), or isotype control antibody (IgG1k; **B**; 1 and 5 µg/ml) for 2 days with human CD150-adapted CDV-A75/17^red^ at a MOI of 0.1. As further control Vero cells expressing canine CD150, which is not recognized by mAb A12, were infected in the absence and presence of mAb A12 (**C**; 1 and 5 µg/ml). Photomicrographs of the autofluorescent virus-encoded tD tomato red were taken under UV light (bar = 100 µm).

To demonstrate that the adaptation occurred to human CD150, and not to another receptor, we performed an infection inhibition experiment using CD150-specific antibodies. Vero-hSLAM cells were incubated with increasing concentrations of anti-CD150 antibody (mAb A12), or control antibody as indicated (Fig. 3) for 3 h prior to infection of cells with human CD150-adapted virus. The infection was dose dependently inhibited with 1 and 5 µg/ml CD150 antibody, but not with control antibody (isotype), indicating that the virus indeed adapted to use human CD150 as receptor. As a further control Vero-cSLAM cells were pre-incubated with mAb A12, which recognizes human CD150 but not canine CD150 (not shown), and subsequently infected. As expected, the antibody does not inhibit the infection of Vero-cSLAM cells.

**Figure 4:**
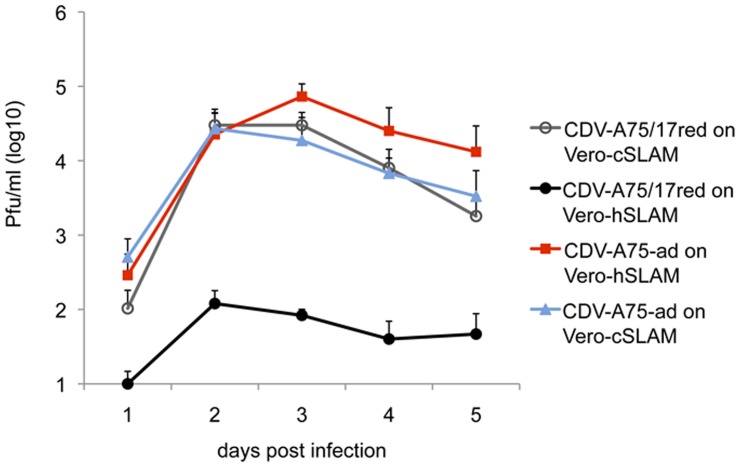
Comparison of single step growth curves of parental and adapted CDV-A75/17^red^ using canine and human CD150-expressing target cells. Vero-cSLAM cells (open circles) and Vero-hSLAM cells (closed circles) were infected with parental CDV-A75/17^red^, and Vero-cSLAM cells (blue triangles) and Vero-hSLAM cells (red squares) were infected with human CD150-adapted CDV-A75/17^red^ (CDV-A75-ad) at a MOI of 0.01 and incubated for indicated times before harvesting cell bound plus released virus. The viruses were then titrated using the optimal target cells for each virus (n = 3, using 3 independently adapted viruses).

Next, single step growth curves of the viruses using various target cells were determined (Fig. 4). Parental wild-type CDV-A75/17^red^ was originally grown using Vero-cSLAM cells. Its growth was reduced by 2–3 logs when Vero-hSLAM cells were infected (MOI = 0.01). In contrast, the growth curve of the human CD150-adapted CDV-A75/17^red^ using these cells (Vero-hSLAM) was similar to the growth of parental CDV-A75/17^red^ using Vero cells expressing dog CD150 (Vero-cSLAM). These results indicate that we obtained complete adaptation of CDV to the human receptor. In addition we determined the growth of human CD150-adapted CDV-A75/17^red^ on canine CD150 expressing cells. Interestingly, the adaptation to human CD150 did not impair growth of this virus on canine CD150 expressing cells.

### Determination of sequence alterations of human CD150-adapted CDV

**Figure 5:**
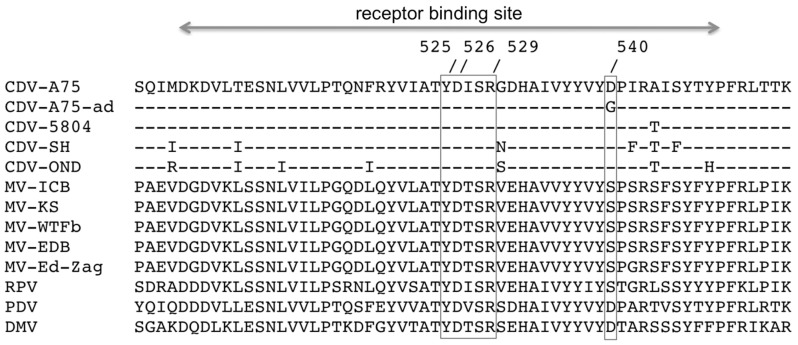
Comparison of the CD150 binding sites of Morbillivirus H proteins. The receptor (CD150) binding site of the Morbillivirus H-proteins comprises approximately amino acid 500 to 550. CDV-H position 540 (corresponding to MV-H position 544) is located nearby the cognate receptor interacting residues 525, 526, and 529 [25]. CDV-A75-ad = human CD150-adapted CDV-A75/17^red^.

As mentioned above, we repeated the adaptation to human CD150 independently three times, and in all trials we observed the same swift adaptation to the human receptor. To determine sequence alterations required for this adaptation, sequences were determined from the original parental recombinant virus and 10 virus clones obtained by plaque picking from the independently adapted viruses. In all sequenced clones of adapted viruses we reproducibly found the same sequence alteration A to G at nucleotide position 8697 in the genome of CDV-A75/17 (AF164967) changing GAC to GGC leading to one expressed amino acid difference in the CDV-H protein at position 540 Asp→Gly (D540G). Amino acid 540 is located within the CD150 binding region of the H protein spanning approximately amino acid 500 to 550 of the CDV-H protein [25]. Aligned sequences of the receptor binding regions of H proteins of CDV-A75/17^red^, human CD150-adapted CDV, other CDV strains, and various Morbilliviruses are shown in Fig. 5.

### Recombinant D540G mutation confers improved interaction with human CD150

**Figure 6:**
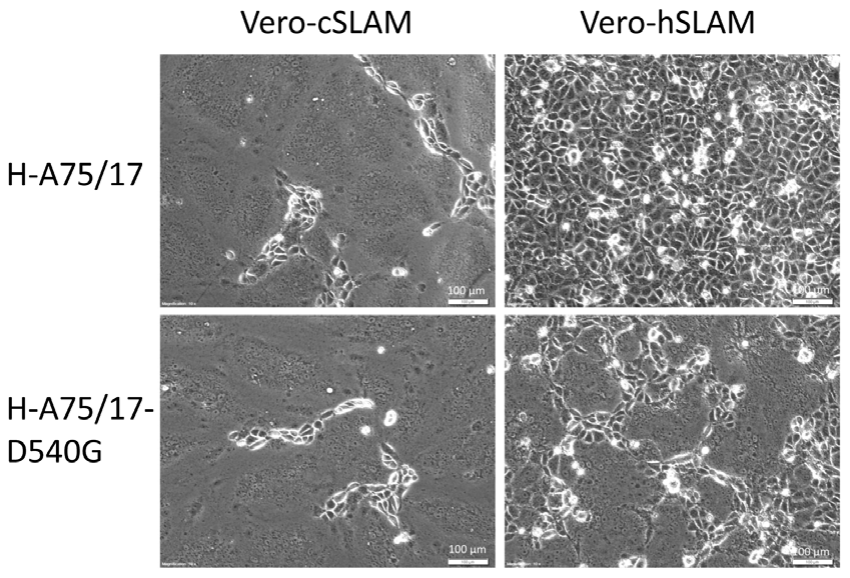
Syncytium formation after co-transfection of plasmids expressing CDV-H and -F proteins. Vero-cSLAM and Vero-hSLAM cells were transfected with plasmids expressing wild-type CDV-H_A75/17_ and mutated H_A75/17-D540G_ in combination with wild-type CDV-F as indicated, and incubated for 24 h before phase-contrast microphotographs were taken (bar = 100 µm).

In order to prove the functional consequences of the detected D540G mutation, we introduced it into an expression vector for CDV-H for transfection experiments. A glycine (G) at position 540 was generated by site directed mutagenesis in the expression plasmid for CDV-H_A75/17_ resulting in pCI-CDV-H_A75/17-D540G_. Vero-cSLAM and Vero-hSLAM cells were transfected with plasmids expressing wild-type H_A75/17_ and F, or expressing the mutated H_A75/17-D540G_ and F. In Vero-cSLAM cells large syncytia were observed at 24 h after transfection with both combinations of plasmids (Fig. 6). In contrast, in Vero-hSLAM cells syncytia were observed only after transfection with plasmids encoding H_A75/17-D540G_ and F, but not after transfection with plasmids encoding for wild-type H and F. This demonstrates that the D540G mutation in CDV-H_A75/17_ is necessary and sufficient to mediate a functional interaction with human CD150 providing full membrane fusion capacity of the viral H and F envelope glycoprotein complex.

### Modelling of the haemagglutinin-CD150 interaction

**Figure 7:**
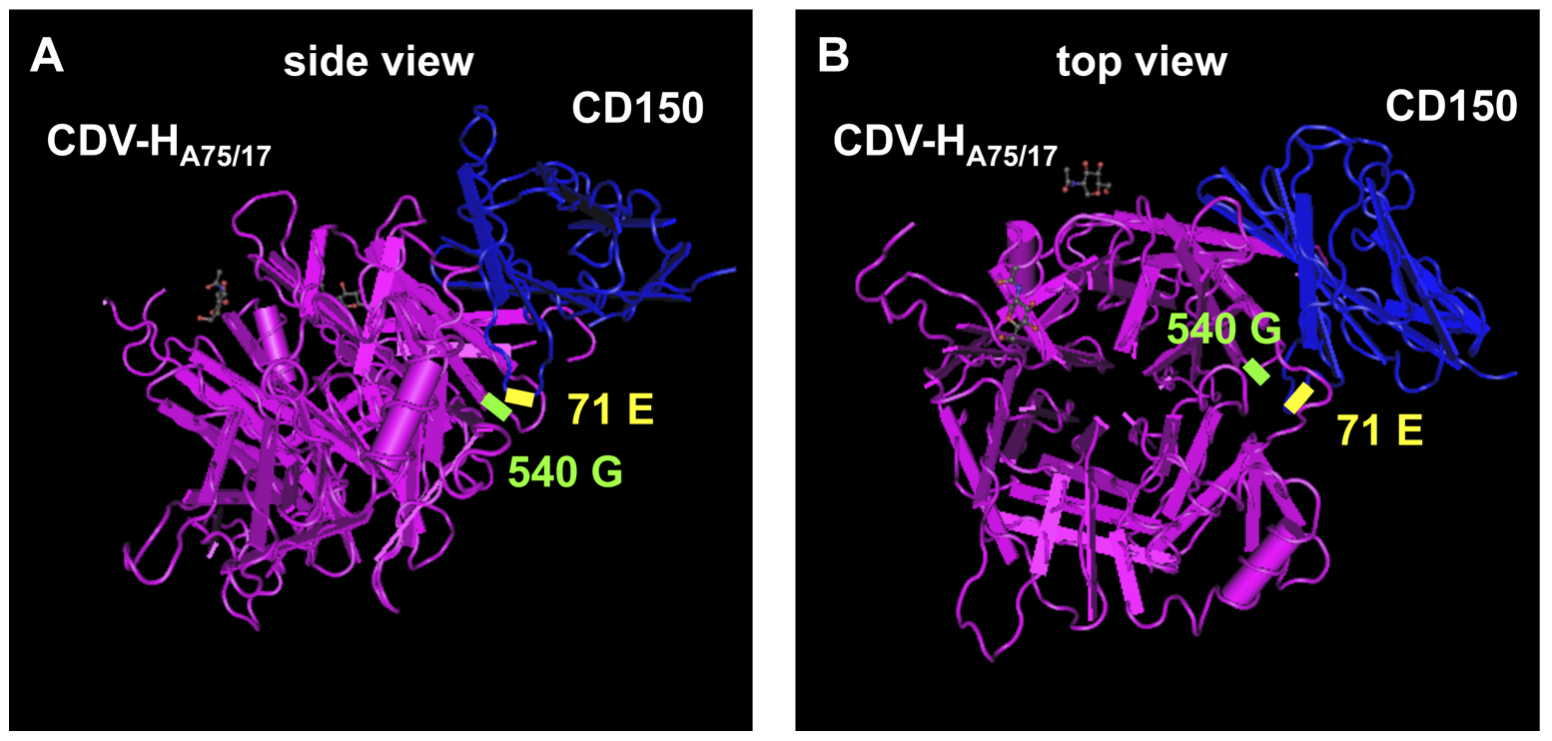
Structural model of the CDV-H-CD150 interaction. Side (A) and top (B) view of the globular head of CDV-H (amino acids 188 to 602) presented in magenta with highlighted amino acid 540 G (green), and of the interacting first V-like domain of human CD150 (amino acids 32 to 140) shown in blue with highlighted amino acid 71 E (yellow). The structures were modelled based on the crystal structure of MV-H bound to CD150 [33].

**Figure 8:**
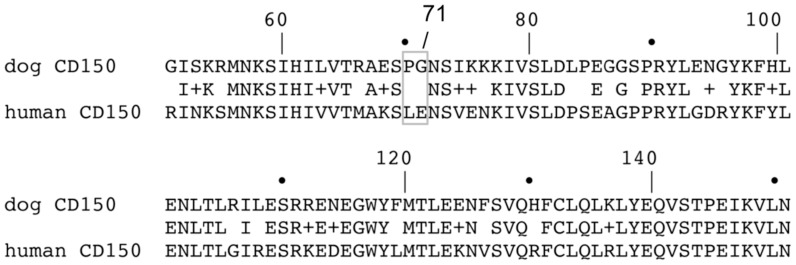
Comparison of the canine and human CD150 sequences around the Morbillivirus H binding site. The MV-H binding site of CD150 was mapped between amino acid 61 and 131 [33]. Amino acid 71 is an uncharged G in dog CD150, and a negatively charged E in human CD150 (NM_001003084 [25]).

Modelling of CDV-H_D540G_ and hCD150 revealed that amino acid 540 in CDV-H is located opposite to a loop in the V-like domain 1 of human CD150 formed by amino acids 70 and 71, Leu and Glu (L and E), respectively (Fig. 7). Interestingly, these two residues differ in canine and human CD150 (Fig. 8). This difference probably exerts the adaptive constraint for amino acid 540 in the interacting CDV-H protein that leads to the observed mutation. The residue at position 71 is a non-charged G in canine CD150, but a charged E in human CD150. It is likely that this charge alteration provoked the adaptive mutation D540G, from a charged to a non-charged residue in the CDV-H protein. Thus, D540G is likely to compensate for the amino acid alteration in the receptor and provides a balanced charge distribution in the CDV-H-receptor complex. Further structural analyses will be undertaken to investigate the molecular interactions leading to this observed adaptive mutation.

## Discussion

Cellular receptors play an important role by controlling the first steps of infection, and, if they are sufficiently different in various hosts, they can provide effective species barriers. Corroborating their similarity in cell tropism and pathogenicity, various Morbilliviruses such as MV, CDV and RPV use the species orthologues of CD150 and nectin-4 as major uptake receptors [16], [17], [26], [27], [28], [29], [30]. Our infection experiment of H358 cells with CDV-A75/17^red^ revealed that few passages were required for adaptation of this virus to human target cells, while the location of the detected mutations outside of H and F clearly indicated that there is no need for adaptation of H to human nectin-4. It is likely that the mutations outside of H and F are required for intracellular adaptation to human host factors, which, however, was not subject of this study and requires further investigations. Nectin-4 is highly conserved between species with almost identical extracellular domains of canine and human nectin-4. Consequently, as observed by us and others [31], no adaptive mutation is required for wild-type CDV to use human nectin-4 as receptor.

With 64% identity, canine and human CD150 are somewhat less conserved. This difference is reflected by our findings that an adaptation phase is required, and that one amino acid exchange at position 540 improves binding to the human receptor. It is astonishing how quickly the adaptation occurred (3 passages), although we started from a cloned recombinant virus with a defined sequence, which was confirmed by sequencing. This parental virus was propagated exclusively using Vero-cSLAM cells before. Given the swift adaptation, it is likely that in a sufficiently large pool of parental virus, as used for initial infection of Vero-hSLAM cells at a MOI of 1.0, the mutation emerges spontaneously. Three passages of “adaptation” are then sufficient to select for the functional H protein. Tatsuo and co-workers investigated earlier the usage of species-specific CD150 as cellular receptors for Morbilliviruses [15]. They analyzed two CDV strains that were isolated using marmoset B95a cells and found that these viruses equally well were taken up by and formed syncytia on CHO cells expressing human or canine CD150. In the light of our results, it is likely that these two CDV strains adapted already during their isolation procedure to marmoset CD150, which is almost identical with human CD150, and therefore can utilize human CD150 very efficiently.

Our selection procedure reproducibly led to viruses bearing the same D540G mutation in the H gene and provided viruses fully adapted to the human receptor. That the observed adaptive mutation is required for a balanced charge distribution as consequence of the alteration in the human CD150 at position 71 appears to be conclusive. This interpretation is supported by the structural modelling data. Our modelling is based on structural analysis of wild-type MV-H interaction with CD150, which revealed the prerequisites for a functional interaction [32]. As shown in Fig. 6, the D540G mutation confers good fusion capacity to the receptor interacting viral envelope proteins, which, however, is not completely as good as observed for the interaction of parental CDV with canine CD150. In spite of this slight difference in the cell-cell fusion, we did not observe a remarkable difference between the titres of these viruses in the single step growth curves. The titre of the adapted virus on Vero-hSLAM cells is even slightly higher at day 3 to 5 (Fig. 4). When syncytia become very large very quickly, membrane parts in the middle may detach, and the amount of newly synthesized virus decreases. Therefore, a slightly reduced fusogenicity and subsequent less detachment from the plastic dish may support a slight increase in virus titre. It was also interesting to observe that the adaptation of CDV-A75/17^red^ to human CD150 did not impair its growth capacity on Vero-cSLAM cells. Although improving the interaction with human CD150, the D540G mutation obviously does not affect the interaction of H with canine CD150. Further structural analyses may clarify this point.

Following the outbreaks of CDV infections in animal facilities in China [5], [7], a CDV outbreak occurred in cynomolgus monkeys (*Macaca fascicularis*) in Japan 2008 [6]. A corresponding CDV strain was isolated (CYN07-dV) and characterized, which efficiently utilized macaca CD150 as entry receptor [6]. Since the amino acid sequence of *Macaca mulatta* CD150 including position 70/71 is practically identical to the human sequence, one may suppose that the human CD150-positive cells should be well infected. In spite of this, the isolated virus did not very well infect Vero-hSLAM cells [6]. The amino acid exchange D540G as observed by us for CDV-A75/17-adaptation was not found in CYN07-dV [6]. Subtle differences between human and macaca CD150 might play a role, such as position 28 (R and H, respectively) and 49 (Y and H, respectively) and contribute to the observed effects.

Taken together, there is obviously no high hurdle for CDV to adapt and utilize human entry receptors. However, in spite of this swift adaptation, these data should not lead to the misunderstanding that other human target cells or humans will be infected so easily. It is clear that adaptation to human entry receptors is only the first step to full adaptation to human target cells, and that alterations in other viral genes are required for intracellular adaptation.
